# Therapeutic ROS and Immunity in Cancer—The TRIC-21 Meeting

**DOI:** 10.3390/cancers13184549

**Published:** 2021-09-10

**Authors:** Sander Bekeschus, Steffen Emmert, Ramona Clemen, Lars Boeckmann

**Affiliations:** 1 ZIK *plasmatis*, Leibniz Institute for Plasma Science and Technology (INP), Felix-Hausdorff-Str. 2, 17489 Greifswald, Germany; ramona.clemen@inp-greifswald.de; 2 Clinic and Policlinic for Dermatology and Venereology, Rostock University Medical Center, Strempelstr. 13, 18057 Rostock, Germany; steffen.emmert@med.uni-rostock.de (S.E.); lars.boeckmann@med.uni-rostock.de (L.B.)

**Keywords:** cold physical plasma, combination therapy, medical gas plasma technology, oncology, reactive oxygen species, RNS, tumor immunology

## Abstract

The first Therapeutic ROS and Immunity in Cancer (TRIC) meeting was organized by the excellence research center ZIK *plasmatis* (with its previous *Frontiers in Redox Biochemistry and Medicine* (FiRBaM) and *Young Professionals’ Workshop in Plasma Medicine* (YPWPM) workshop series in Northern Germany) and the excellence research program ONKOTHER-H (Rostock/Greifswald, Germany). The meeting showcased cutting-edge research and liberated discussions on the application of therapeutic ROS and immunology in cancer treatment, primarily focusing on gas plasma technology. The 2-day hybrid meeting took place in Greifswald and online from 15–16 July 2021, facilitating a wide range of participants totaling 66 scientists from 12 countries and 5 continents. The meeting aimed at bringing together researchers from a variety of disciplines, including chemists, biochemists, biologists, engineers, immunologists, physicists, and physicians for interdisciplinary discussions on using therapeutic ROS and medical gas plasma technology in cancer therapy with the four main sessions: “Plasma, Cancer, Immunity”, “Plasma combination therapies”, “Plasma risk assessment and patients studies”, and “Plasma mechanisms and treated liquids in cancer”. This conference report outlines the abstracts of attending scientists submitted to this meeting.

## 1. Introduction

Reactive oxygen species (ROS) have critical functions in cell signaling, cell damage, immune response, and diseases. In cancers, ROS generating therapies promote beneficial effects on the inactivation of tumor cells in vitro and in vivo, as has been evident for decades for photodynamic therapy and, more recently, medical gas plasma technology. Sixty-six participants attended 35 oral presentations within four main sessions in this first *Therapeutic ROS and Immunity in Cancer* (TRIC) meeting in 2021 (TRIC-21). The international TRIC-21 meeting was a joint international meeting of the excellence research center ZIK *plasmatis* (with its previous Frontiers in *Redox Biochemistry and Medicine* (FiRBaM) and *Young Professionals’ Workshop in Plasma Medicine* (YPWPM) workshop series in Northern Germany) and the excellence research program ONKOTHER-H (Rostock/Greifswald, Germany). The main topics of the conference included “Plasma, Cancer, Immunity”, “Plasma combination therapies”, “Plasma risk assessment and patients studies”, and “Plasma mechanisms and treated liquids in cancer”.

The meeting participants presented a series of ground-breaking studies, with nearly half of them involving in vivo research. This clearly shows the field expanding to more clinically relevant models to identify promising research avenues to transit clinical plasma oncology into patient applications. While this is a long-term goal, key points were identified that help the transition of the in many cases laboratory-based devices towards standardized application settings, ultimately involving standardized manufacturing, patenting, engagement of companies, regulatory affairs, and ultimately the interest and enthusiasm of bringing devices into phase I safety clinical trials as a prerequisite for any further efficacy testing. For example, while the atmospheric pressure argon plasma jet kINPen MED (neoplas MED GmbH, Greifswald, Germany) has already entered small-scale clinical studies in oncology and for treating dozens of precancerous and cancerous lesions in hundreds of treatment sessions thanks to the passion of two key medical doctors in the field of plasma oncology, Hans-Robert Metelmann and Christian Seebauer, there are new initiatives at the horizon in Canada and the U.S. to promote this route with other jet-based devices further. To this end, the safety and risk assessment session of our meeting was a significant contribution to such considerations.

The organizers gratefully acknowledge two EUR 100 cash prizes sponsored by HIDEN ANALYTICAL for the best presentations. The first prize was given to Audrey Glory from the group of Prof. Philipp Wong for her excellent and much-focused contribution on using a novel jet-based device for in vitro and in vivo mono and combination breast cancer treatment, and for taking part in a bigger team preparing first clinical experience of this device in the field of oncology in Montreal, Canada. The second prize was given to Julia Berner. She works under the supervision of Hans-Robert Metelmann, Christian Seebauer, and Sander Bekeschus within the ZIK *plasmatis* and ONKOTHER-H excellence programs on deciphering the immuno-relevant and molecular signatures of adaption of head and neck cancer cells towards multiple cycles of plasma treatment. Moreover, the meeting would not have been possible without the contribution of the Alfried Krupp Wissenschaftskolleg Greifswald, Germany, and its academic coordinator Christian Suhm, for providing technical and organizational support and realizing the funding of the conference report. The scientific committee also wishes to thank Tobias Fischer (Clinic and Policlinic for Dermatology and Venereology, Rostock University Medical Center, Rostock, Germany) for administrative support and organization of the TRIC-21 meeting.

In the following, the meeting abstracts of scientists are listed that participated in the meeting in alphabetical order. The presenting authors are underlined.

## 2. Conference Abstracts

### 2.1. Challenges and Opportunities of Gas Plasma Technology in Oncology and Immunology

BekeschusSandervon WoedtkeThomasWeltmannKlaus-DieterWendeKristianZIK *plasmatis*, Leibniz Institute for Plasma Science and Technology (INP), Greifswald, Germany

It has been 15 years since the first report on the ability of gas plasma to inactivate cancer cells and 10 years since the first promising results in animal experiments. Now, it is time to review the past decade’s progress and pinpoint promising therapeutic routes in oncology using gas plasma technology. This relates to the mechanisms of action elicited by the gas plasma-derived ROS/RNS in cancer cells and the action and propagation of the immune system and immune responses, respectively. Another promising route is combination therapies using gas plasma technology along with novel and established oncological routines. Special emphasize will be given to the role of the immune system, particularly the immunogenic cancer cell death, observed in several in vitro and in vivo studies performed with different plasma devices. Finally, considerations about the safety and types of tumors will be outlined.

**Funding:** The work of Sander Bekeschus and his team was or is funded by the German Federal Ministry of Education and Research (grant numbers 03Z22DN11, 03Z22Di1, 16GW0344K, 03COV06A, and 03Z22D511), the European Social Fund (ESF, grant number ESF/14-BM-A55-0006/18), the German Research Foundation (DFG, grant number AOBJ 669606), the Comprehensive Cancer Center (CCC) Mecklenburg-Western-Pomerania (Germany), the Ferdinand-Eisenberger-Stipendium in Urology (Germany), and the Stiftung Tumorforschung Kopf-Hals (Germany).

### 2.2. Adaptive Responses of Head and Neck Cancer Cells upon Repeated Exposure to Gas Plasma over Ten Weeks In Vitro?

BernerJulia^1^,^2^SeebauerChristian^1^MetelmannHans-Robert^1^BekeschusSander^2^1Department of Oral, Maxillofacial Surgery, and Plastic Surgery, Greifswald University Medical Center, Greifswald, Germany2ZIK *plasmatis*, Leibniz Institute for Plasma Science and Technology, Greifswald, Germany

For several years, physical plasma has been used in various medical fields such as wound healing, blood coagulation, and dentistry. Due to its promising anti-tumoral and immunogenic properties as well as negligible side effects, physical plasma also gained relevance as an innovative cancer treatment tool over time. First, clinical studies tested its application as an adjuvant therapy to head and neck cancer (HNC), the sixth most common malignancy worldwide. Although the trials indicated a successful treatment, they were partly followed by a potential relapse of tumor growth. For further optimization of physical plasma as a complementary cancer treatment, investigations concerning the tumor microenvironment, the genetic equipment, and the mode of action of plasma are needed. Therefore, we established a new in vitro cell culture model where we treated two different HNC cell lines repetitively once per week with plasma over nine weeks. This enabled us to follow up on transcriptomic changes and alterations of the viability and secretion profiles of the cancer cells upon continuous plasma treatment. Thus, it helped us identify proteins that mediate the plasma-related effects within the cancer cell and might cause a more or less efficient impact of the plasma therapy in head and neck cancer.

**Funding:** This research was funded by the European Social Fund (ESF) and the Ministry of Education, Science, and Culture of Mecklenburg-West Pomerania, Germany, grant numbers ESF/14-BM-A55-0005/18 and ESF/14-BM-A55-0006/18, as well as the German Federal Ministry of Education and Research (BMBF), grant numbers 03Z22DN11 and 03Z22Di1.

### 2.3. Assessment of Cold Atmospheric Pressure Plasma as Innovative Therapy for Treatment of Radiation Dermatitis Using a MOUSE Model

BernhardtThoralf^1^MandaKatrin^2^HildebrandtGuido^2^StachsOliver^3^BekeschusSander^4^VollmarBrigitte^5^EmmertSteffen^1^BoeckmannLars^1^1Clinic and Policlinic for Dermatology and Venereology, University Medical Center Rostock, Rostock, Germany2Clinic and Policlinic for Radiation Therapy, University Medical Center Rostock, Rostock, Germany3Department of Ophthalmology, University Medical Center Rostock, Rostock, Germany4ZIK *plasmatis*, Leibniz Institute for Plasma Science and Technology (INP), Greifswald, Germany5Rudolf-Zenker-Institute for Experimental Surgery, University Medical Center Rostock, Rostock, Germany

A substantial number of cancer patients receiving radiotherapy develop radiation dermatitis leading to erythema, edema, moist desquamation, and ulceration. It usually is accompanied by pain and strong pruritus, which may lead to an interruption or even to an abortion of the therapy. As cold atmospheric pressure plasma (CAP) supports wound healing and regenerative processes without causing any relevant side effects, we hypothesized CAP treatment to reduce the severity of radiation dermatitis allowing an uninterrupted radiotherapy. Hence, this study aimed to assess the clinical course and the molecular pathomechanisms of radiation dermatitis and their modulation by treatment with CAP. For this purpose, an acute radiation dermatitis was induced in nude mice. A scoring system and noninvasive imaging techniques such as hyperspectral imaging, laser scanning microscopy, and optical coherence tomography enabled the monitoring of the course of the disease. After identifying the optimal radiation dose (65 Gy) for inducing a moderate radiation dermatitis (score 2.5) using a gamma irradiator, we assessed the efficacy of CAP in treating such a moderate radiation dermatitis using an atmospheric pressure plasma jet in comparison to an untreated control group. In addition, skin biopsies from CAP treated and untreated mice were collected for immunohistochemistry and transcriptome analyses.

**Funding:** This research was funded by the Damp Foundation grant no. 2017-05.

### 2.4. Cold Atmospheric Plasma for Modulation of Skin Normal and Cancer Cells ^§^

BhartiyaPradeep^1^KaushikNeha^2^NguyenLinh Nhat^1^BekeschusSander^3^MasurKai^3^KaushikNagendra Kumar^1^ChoiEun Ha^1^1Plasma Bioscience Research Center, Kwangwoon University, Seoul, Korea2Department of Biotechnology, College of Engineering, Suwon University, Hwaseong, Korea3ZIK *plasmatis*, Leibniz Institute for Plasma Science and Technology (INP), Greifswald, Germany§This contribution was accepted but not part of the oral program.

In the most recent decade, cold atmospheric plasma (CAP) technology has bloomed into a safe and agreeable anti-cancer therapeutic module and must be explored extensively for a deeper understanding. Several studies have shown that CAP can selectively attenuate cancer cells and stimulate normal cells by inducing oxidative stress-dependent pathways. There is growing need for utilization of plasma technology not only for cancer attenuation but also for fundamental functions and molecular interactions of cells against oxidative stress. Skin is mostly exposed to environmental insults and exogenous factors. While plasma technology has been utilized greatly for wound healing and studied for melanoma inhibition, there is a lack of understanding of molecular interactions in skin cells. In this study, we aim to study molecular changes within cells when exposed to plasma. We are studying two objectives: (i) decipher the microRNA regulation network in melanoma cells aiding in plasma-based cancer attenuation and (ii) to understand the subtle changes in normal cells due to repetitive plasma exposure over long time. We established the plasma conditions required for melanoma (SK-2) cells inhibition in vitro and investigated changes in dermal fibroblast (GM00637) cells over period of 12 weeks.

**Funding:** This research was funded by the National Research Foundation under the Ministry of Science and ICT (2021R1A6A1A0303878511), (NRF-2021R1C1C1013875), and (2021R1F1A105569411) of the Korean Government.

### 2.5. Clinical Studies in Plasma Medicine and Safety Considerations

BoeckmannLarsSchäferMirjamThoralfBernhardtMannMiriamEmmertSteffenClinic and Policlinic for Dermatology and Venereology, University Medical Center Rostock, Rostock, Germany

The field of plasma medicine is receiving increasing attention and is moving steadily from bench to bedside. While the application of cold atmospheric pressure plasma (CAP) for wound treatment has already moved successfully from basic research into clinical practice, the use of CAP for cancer treatment is still in the process of moving from preclinical research to first clinical studies. An important aspect in this translational process is to rule out safety concerns regarding the CAP producing devices as well as the cold plasma itself. The use of many different devices or modifications by different research groups pose a challenge with respect to comparability of the results obtained with these devices, leading to difficulties defining long-term safety and efficacy of plasma devices in a comparable and standardized manner. In order to establish general requirements for plasma sources in medicine, a DIN SPEC 91315 was published in 2014 in Germany. This standard is currently developed further into a DIN Norm. In this presentation, I will discuss clinical studies for CAP application in wound healing and cancer treatment alongside with standardization and safety consideration.

**Funding:** The work of Lars Boeckmann and colleagues is funded by the German Federal Ministry for Economic Affairs and Energy (grant number 03TN0019B), the German Federal Ministry of Education and Research (16GW0345), the German Research Foundation (EM 63/13-1), and the Damp Foundation (2017-05). Furthermore, the joint research project “ONKOTHER-H” is supported by the European Social Fund (ESF), reference: ESF/14-BM-A55-0001/18 and the Ministry of Education, Science and Culture of Mecklenburg-Vorpommern, Germany.

### 2.6. Safety and Cellular Response Pathways to Reactive Species in Plasma Activated Liquids

BoehmDaniela^1^TsoukouEvanthia^1^LopesBeatriz Pinheiro^1^BourkePaula^1^,^2^,^3^1Environmental Sustainability and Health Institute and School of Food Science and Environmental Health, Technological University Dublin, Dublin, Ireland2Plasma Research Group, School of Biosystems and Food Engineering, University College Dublin, Dublin, Ireland3School of Biological Sciences, Queens University Belfast, Belfast, Northern Ireland, United Kingdom

Plasma activated liquids (PAL), generated by exposing liquids to different plasma discharges, can retain longer-lived plasma reactive species and have demonstrated biological activity, including cytotoxic effects. The reactive species composition depends on plasma device, discharge parameters and liquid target characteristics but the characterization of these liquids is often limited to the detection of a selection of stable and easily detectable reactive oxygen and nitrogen species (RONS) such as hydrogen peroxide, nitrite, and nitrate. Our work has focused on elucidating the effects of distinct chemical profiles in PAL (ROS- or RNS-dominated) on different biological systems and establishing the underlying mechanisms of action. Simple plasma activated solutions (water, saline, and buffer) and more complex model biomolecule solutions were investigated to establish the interconnection of antioxidant status, intracellular ROS, mitochondrial membrane depolarization, and induction of apoptotic/necrotic cell death pathways as a function of reactive species composition and concentration. Detailed understanding of cellular responses and cytotoxic effects of plasma reactive species provides a basis for the development of plasma and/or PAL in oncology as stand-alone interventions or in combination with other approaches such as chemotherapeutics, prodrugs or nanoparticles where they may be employed as delivery/activation mechanisms or for synergistic effects.

**Funding:** This research is supported by funding from Science Foundation Ireland under grant number 15/SIRG/3466 and the Irish Research Council under the Enterprise Partnership Scheme.

### 2.7. Pin Electrode Reactor: A Novel Cold Atmospheric Plasma Device and Its Potential in Glioblastoma Treatment

CarvalhoAndressa Maria Aguiar de^1^,^2^,^3^ScallyLaurence^1^,^6^CullenPatrick J.^1^,^5^,^6^TiwariBrijesh^2^CurtinJames F.^1^,^4^1BioPlasma Research Group, School of Food Science and Environmental Health, Technological University Dublin, Dublin, Ireland2Department of Food Biosciences, Teagasc Food Research Centre, Ashtown, Dublin, Ireland3Environmental Sustainability & 3Health Institute (ESHI), Technological University Dublin, Dublin, Ireland4FOCAS Research Institute, Technological University Dublin, Dublin, Ireland5University of Sydney, School of Chemical and Biomolecular Engineering, Sydney, Australia6Plasmaleap Technologies, Merewether Building, City Road, Sydney, Australia

Glioblastoma (GBM) is the most malignant and frequent type of brain tumor. The current standard therapy for this disease consists of surgical resection, followed by radiotherapy and chemotherapy. Prognosis is poor, with a life expectancy of fourteen months after diagnosis. Treatment is limited due to the area for the surgical resection and for the inability of some drugs to cross the brain blood barrier. Cold atmospheric plasma (CAP) is a new approach in the treatment of this challenging disease. Considering the plasma self-adaptation that different plasma discharge modes can undergo, which leads to different interaction plasma/cells, the characterization of a new device is essential. In this study we analyzed the effect of a novel large pin-to-plate non-thermal atmospheric plasma on U-251 MG cells under different conditions. The analysis of reactive oxygen and nitrogen species (RONS) on plasma, media and cells were also assessed. We were able to demonstrate that the pin-to plate device is cytotoxic to GBM cells in a dose, time, and ROS dependent manner. The measurements of RONS on plasma/media also give us an insight on the chemical effect of this novelty device, and the possibility to better understand the use of this device as a promising GBM therapy.

**Funding:** This research was funded by Science Foundation Ireland Grant Numbers 14/IA/2626 (P.C., J.C.) 17/CDA/4653 (A.C., B.T., P.C., and J.C.).

### 2.8. Transdermal Cold Atmospheric Plasma-Mediated Immune Checkpoint Blockade Therapy

ChenGuojun^1^,^2^ChenZhitong^3^,^4^WirzRichard E.^3^GuZhen^1^,^5^1Department of Bioengineering and the Rosalind & Morris Goodman Cancer Research Centre, University of California-Los Angeles, Los Angeles, USA2Department of Biomedical Engineering, McGill University, Montreal, Canada3Department of Mechanical and Aerospace Engineering, University of California-Los Angeles, Los Angeles, USA4National Innovation Center for Advanced Medical Devices, Shenzhen, China5College of Pharmaceutical Sciences, Zhejiang University, Hangzhou, China

Immune checkpoint blockade (ICB) therapy increases antitumor immune responses by inhibiting intrinsic down-regulators of immunity, and has greatly transformed the landscape of cancer therapeutics. However, the overall objective rate of ICB remains modest, and ICB is often associated with severe side effects. Hence, strategies to improve efficacy and reduce side effects of ICB therapy are clinically relevant. Here, we reported a microneedle-integrated cold atmospheric plasma (CAP)-mediated approach for enhanced ICB therapy. Leveraging microneedles with a unique hollow structure, CAP can be effectively delivered through the skin, interacting with the tumor tissue. The resulting tumor-associated antigen presentation by dendritic cells and the following T cell-mediated immune response augmented by immune checkpoint inhibitors released from the microneedles further boost anticancer immunity locally and systemically. In melanoma-bearing mice, the transdermal combined CAP and ICB therapy can inhibit the tumor growth of both primary tumors and distant tumors, prolonging the survival of mice.

**Funding:** This work was supported by grants from the start-up packages of University of California, Los Angeles (UCLA), NIH (R01 CA234343-01A1), Air Force Office of Scientific Research (FA9550-14-10317, UCLA Subaward No. 60796566-114411), and Jonsson Comprehensive Cancer Center at UCLA.

### 2.9. The Effects of Atmospheric-Pressure Cold Plasma Generated Short-Life Species and Long-Life Species on Skin Cancer Cells

ChienPo-Chien^1^ChenChao-Yu^1^SatoTakehiko^2^ChengYun-Chien^1^1Department of Mechanical Engineering, National Chiao Tung University2Institute of Fluid Science, Tohoku University

We investigated the effects of atmospheric-pressure cold plasma-generated short-lived species (such as •OH radicals), long-lived species, and electric fields on skin cancer cells (melanoma, A2058, and basal cell carcinoma, BCC) and normal cells (BJ cells, Detroit 551 cells). The distance between the cells and plasma–medium interface was fine tuned to treat the cells with different concentrations of short-lived species especially •OH. The cell viability and apoptosis were compared to determine which factor had a greater impact on the cells. The Annexin V and PI stain was used check the cell apoptosis and death. With our experimental setup, long-lived species and electric field generated by the plasma did not have significant effects on either the normal or cancer skin cells. In contrast, the short-lived species significantly inhibited the viability and induced apoptosis of skin cancer cells but not the normal skin cells. This study verified that short-lived species in plasma inhibit skin cancer cells more than normal skin cells. In the future, more cell lines will be tested with this experiment setup to check the effects of short-lived species. This work can verify the importance of plasma in clinical application. Moreover, ROS adjustment of plasma can be further optimized.

**Funding:** Ministry of Science and Technology, Taiwan for the financial support (Grant number: 109-2221-E-009-016-MY2). The Collaboration Research Project of the Institute of Fluid Science, Tohoku University, Japan (Project code: J20I083).

### 2.10. Gas Plasma-Medicated Oxidative Modifications in Immunobiology and Cancer Treatment

ClemenRamonaBekeschusSanderZIK *plasmatis*, Leibniz Institute for Plasma Science and Technology (INP), Greifswald, Germany

Proteins with post-translational modifications (PTMS) are vital factors in disease development and target disease control through use as an optimized vaccine. Intracellular proteins become fragmented to be presented as tolerated or immunogenic peptides on the major histocompatibility complexes on the cell surface. T-cells recognize the antigenic peptides and differentiate between self- and non-self-structure to either induce or prevent an immune response. To investigate the immunogenicity of oxidative PTMs in antigenic peptides, we treated chicken hen egg white protein (Ovalbumin) with cold physical plasmas. These highly reactive, partially ionized gases simultaneously generate various reactive oxygen and nitrogen species, making them ideal tools for studying many oxPTMs, such as chlorination, quinone, and oxidation. We also observed modifications of the tertiary structure of Ovalbumin after plasma treatment. The immunogenicity of the oxPTMs and structural changes was tested by re-challenging mice genetically engineered to generate Ovalbumin-specific T cells (OT-II). Plasma-treated Ovalbumin (oxOva) increased the number of CD25+/CD69+ activated CD4+ T cells ex vivo and in vivo. Wild-type mice without Ovalbumin-specific T-cells were vaccinated and re-challenged with native Ova or oxOva. With the T-cell activation being 1.5-fold, vaccination and rechallenge with oxOva were more efficient than native OVA. We conclude that cold physical plasmas-induced PTMs improve the immunogenic properties of Ovalbumin.

**Funding:** This research was funded by the German Federal Ministry of Education and Research, grant numbers 03Z22DN11 and 03Z22Di1.

### 2.11. Synthesis of Small Molecules for Skin Cancer Treatment with Plasma- and Non-Plasma Application

FreyAnnaStillerTanjaHeinMartinLangerPeterInstitute of Chemistry, University of Rostock, Rostock, Germany

As part of the joint project ONKOTHER-H, our subproject focuses on the synthesis of novel small molecule drugs with potential for plasma- and non-plasma applications in anti-melanoma therapy. Hence, it was possible to synthesize and fully characterize interesting candidates, such-as N-glycosylated isatines bearing withdrawing substituents (F, Cl, Br) as building blocks for N-glycosylated oxindoles as well as indirubin- and sulfur-analogous indirubin-N-glycosides. In addition, further useful small molecules as bis-ullazines and dibenzotropanes were synthesized. Particularly, the synthesis of bis-ullazines seems to be promising for the combination with cold atmospheric plasma due to the formation of stable aromatic fragments. Furthermore, interesting candidates were found by in vitro screening. In the future, these substances will also be analyzed through in vivo tests.

**Funding:** This joint research project “ONKOTHER-H” is supported by the European Social Fund (ESF), reference: ESF/14-BM-A55-0004/18, and the Ministry of Education, Science and Culture of Mecklenburg-Vorpommern, Germany.

### 2.12. Broadening the MHC Class 1 Epitope Repertoire with Non-Thermal Plasma to Confer Protective Immunity against Adult T-Cell Leukemia

GinwalaRashida^1^MulherkarRia^1^MohamedHager^1^KarabudakAykan^2^HuangXiaofang^2^RowanAileen^3^PhilipRamila^2^KrebsFred^1^KhanZafar K.^1^MillerVandana^1^JainPooja^1^1Department of Microbiology and Immunology, Drexel University College of Medicine, Philadelphia, PA, USA2Emergex, Pennsylvania Institute for Biotechnology, Doylestown, PA, USA3Department of Medicine, Imperial College, London, UK

Adult T-cell leukemia/lymphoma (ATLL), caused by human T-cell leukemia virus type 1 (HTLV-1) infection, is a rare and aggressive disease with poor clinical prognoses because of chemoresistance, associated immunosuppression, and T-cell exhaustion. Patients with chronic HTLV-1 infections have high frequencies of HTLV-1-activated CD8+ T cells and have predominantly two HLA alleles (A2, A24). Our studies utilizing an immunoproteomics approach to characterize MHC-I restricted epitopes presented by HLA-A2+, A24+, MT-2, and SLB-1 cell lines showed that six novel MHC-I restricted epitopes bound to HLA-A2 and HLA-A24 alleles. After generating CD8+ T cells specific for each of these peptides, MagPix MILLIPLEX analyses showed that these cells secreted IFN-γ, granzyme B, MIP-1α, TNF-α, perforin, and IL-10 when cultured in the presence of MT-2 cell line and showed cytotoxic activity when co-cultured with MT-2 cells. A CD8+ T-cell killing assay indicated significant antiviral activity of CD8+ T cells specific to all identified peptides. Future efforts to develop vaccination strategies for ATLL will explore the use of oxidative stress-inducing non-thermal plasma (NTP) to broaden the array of MHC I viral peptides displayed on infected cells, as we have previously demonstrated in studies involving T cells expressing the human immunodeficiency virus type 1 (HIV-1) provirus.

**Funding:** This research was supported by NIH 5R01NS097147-04. This research was also facilitated by a travel grant provided by the Global Engagement Funding program of the Drexel University Office of Global Engagement and Education Abroad, as well as funding from the Department of Microbiology and Immunology and the Institute for Molecular Medicine and Infectious Disease at the Drexel University College of Medicine.

### 2.13. On the Selectivity of Plasma Treatment In Vitro Due to Short- and Long-Lived RONS

SkliasKyriakos^1^SousaJoão Santos^1^GirardPierre-Marie^2^,^3^1Université Paris-Saclay, CNRS, Laboratoire de Physique des Gaz et des Plasmas, Orsay, France2Institut Curie, PSL Research University, CNRS, INSERM, UMR 3347, Orsay, France3Université Paris-Saclay, CNRS, UMR 3347, Orsay, France

Over the last few years, plasma-activated liquids (PAL) have gained interest for their anti-cancer properties. Two treatment modalities can be applied to the cells, direct and indirect plasma treatments. For direct plasma treatment, the cells covered by a liquid (e.g., cell culture medium or saline buffered solution such as PBS) are present during the treatment time (phase I, plasma ON) and the incubation time (phase II, plasma OFF), while for indirect plasma treatment, the cell-free liquid is firstly plasma-treated and then applied to the cells. The scope of our work was to study these two treatment modalities to bring new insights into the potential use of PAL for cancer treatment. We provide strong evidence that, in vitro, the concentration of RONS (H_2_O_2_, NO_2_^−^ and NO_3_^−^) in combination with the acidic pH are the main drivers of plasma-induced PBS toxicity in tumor cells but not in normal cells, which makes ad hoc reconstituted solutions powerful anti-tumor treatments. In marked contrast, direct plasma treatment is deleterious for normal cells in vitro due to the synergy between short- and long-lived reactive species. In light of our results, I will discuss the limitations of PAL application on cancer treatment.

**Funding:** This work was financially supported by the LabEx LaSIPS (project PHeCell3d) and the Université Paris-Saclay (Strategic Research Initiative NanoTheRad) and performed in the framework of the CNRS network GdR 2025 HAPPYBIO.

### 2.14. SCRIPT: A Novel Way to Improve the Local Control of Breast Cancers

GloryAudrey^1^LafontaineJulie^2^BoisvertJean-Sébastien^1^,^2^CoulombeSylvain^2^WongPhilip^1^,^3^1Institut du cancer de Montreal, CR-CHUM, Montreal, Quebec, Canada2Department of Chemical Engineering, McGill University, Montreal, Quebec, Canada3Department of Radiation Oncology, Princess Margaret Cancer Center, Toronto, Ontario, Canada

The treatment of many cancers involves multimodal approaches that combines different therapies to improve clinical outcomes. In curative breast cancer, the standard of care often involves breast conservative surgery, with the choice of systemic treatment being influenced by the cancer’s subtype and adjunctive radiotherapy (RT). To further improve the outcome of breast cancer patients, we developed the SCRIPT project: Surgery Combined with Radiotherapy and Intra-operative Plasma Treatment. Our non-thermal plasma (NTP) device is a radiofrequency plasma jet generating plasma by applying an oscillating electric field (13.56 MHz) to a flow of helium, passing through electrodes in a coaxial configuration. The first milestone was to test the combination of NTP and RT in vitro on seven breast cancer cell lines, showing a good correlation in sensitivity between the two treatments and a synergistic effect in some cell lines. These results encouraged us to pilot an in vivo study using the triple negative breast cancer MDA-MB-231 murine xenograft to evaluate NTP, alone or in combination with RT, in delaying tumor growth. The combination of plasma and RT significantly reduced tumor growth compared to non-irradiated tumors. Judicious use of RT and NTP is a strategy that shows potential for clinical translation.

**Funding:** This research was funded by MEDTEQ, grant number 8G (including contributions from NexPlasmaGen Inc. and InstaDesign Dev.). J.-S.B. and A.G. are supported by Mitacs, grant number #IT10966. J.-S.B. is supported by TransMedTech, NSERC and FRQNT. Additionally, P.W. is supported by the FRQS grant numbers 32730 and 34612.

### 2.15. Nonthermal Biocompatible Plasma for Immunomodulation

KaushikNagendra Kumar^1^BhartiyaPradeep^1^RanaJuie^1^NguyenLinh Nhat^1^KaushikNeha^2^ChoiEun Ha^1^1Kwangwoon University, Seoul, Korea2Suwon University, Hwaseong, Korea

The application of plasma medicine technology has been actively explored over the last decade. Recently, non-thermal plasmas have demonstrated their potential as a safe anticancer therapeutic approach that can kill various types of cancer targets. There is an urgent need for new human health care technology against cancers based on immuno-modulation. Our research work mainly comprises plasma-induced activation of immune cells or systems, which finds applications for curing various kinds of resistant tumors and other dreadful diseases. Our main objectives are (i) to clarify the basic mechanism on plasma-induced immune-modulations; (ii) to develop an immunomodulation-based strategy for the treatment of various dreadful diseases including cancers; and (iii) to perform a pre-clinical and standardization study. A recent preliminary study suggests that plasma significantly modulates immune cells and can induce cancer cell death in co-culture conditions. Recently, we have reported that plasma-modulated monocytes and cytotoxic macrophages release TNF-α, and other relevant cytokines which block cancer cell growth and can have the potential to contribute to reducing tumor growth in patients soon. Additionally, we have investigated plasma-induced molecular patterns to activate the cancer immunity cycle for cancer treatment. Recently, we also have investigated a few plasma treatment strategies to inactivate coronaviruses.

**Funding:** This research was funded by the National Research Foundation under the Ministry of Science and ICT (NRF-2016K1A4A3914113), (NRF-2021R1C1C1013875) of the Korean Government.

### 2.16. Evaluation of the Anticancer Effect of Cold Atmospheric Pressure Plasma on Skin Cancer by Multimodal Imaging Techniques

KordtMarcel^1^VollmarBrigitte^1^GrambowEberhard^1^,^2^1Rudolf-Zenker-Institute of Experimental Surgery, Rostock University Medical Center, Rostock, Germany2Department for General, Visceral-, Vascular- and Transplantation Surgery, Rostock University Medical Center, Rostock, Germany

Skin cancer represents the most common form of cancer worldwide and is commonly divided into non-melanoma skin cancer (NMSC) including basal cell carcinoma (BCC) as well as squamous cell carcinoma (SCC) and malignant melanoma (MM). This study aimed to evaluate the effects of cold atmospheric pressure plasma (CAP), one of the most promising approaches to cancer therapy in recent years, on SCC and MM in vivo by means of a comprehensive approach using multimodal imaging techniques. Longitudinal MR and PET/CT imaging were performed to determine the anatomic and metabolic tumor volume over 3-weeks in vivo, formation of reactive species after CAP treatment was assessed with the non-invasive luminescence measurement of L-012. Histology, immunohistochemistry, and Western Blot analysis were used to study proliferation and apoptosis in CAP-treated tumors. The results showed substantial inhibition of tumor growth and increase of reactive species after CAP treatment over the observation time in comparison with untreated tumors. In summary, it can be assumed that CAP represents a promising treatment option for cancer therapy and offers the possibility of treating topically localized tumors. Thus, therapy with CAP could be added as a possible adjuvant therapy option in addition to established standard therapies for skin cancer.

**Funding:** This joint research project ONKOTHER-H is supported by the European Social Fund (ESF/14-BM-A55-0003/18) and the Ministry of Education, Science and Culture of Mecklenburg-Vorpommern, Germany.

### 2.17. Scientists Reflect on Animal Testing—Guideline Interviews with Following Qualitative Content Analysis

Kwiatek-ScholzElisa^1^SuhmChristian^2^BekeschusSander^3^FischerTobias^4^VollmarBrigitte^5^MetemannHans-Robert^1^WitzkeKatharina^1^1Department of Oral and Maxillofacial Surgery/Plastic Surgery, Greifswald University Medicine, Greifswald, Germany2Alfried Krupp Wissenschaftskolleg Greifswald, Greifswald, Germany3ZIK *plasmatis*, Leibniz Institute for Plasma Science and Technology (INP), Greifswald, Germany4Department of Dermatology and Venereology, Rostock University Medicine, Rostock, Germany5Rudolf-Zenker-Institute for Experimental Surgery, Rostock University Medicine, Rostock, Germany

Scientists use animal testing to develop tumor treatment methods. They are also performed in the ONKOTHER-H research project to investigate the mode of action of small molecules and cold atmospheric pressure plasma on tumors. The use of animal testing is constantly discussed ethically. Therefore, the ONKOTHER-H research project evaluates ethical aspects of animal ethics in the development of innovative tumor treatment methods in a sub-project. It examines how scientists that are working with animal testing reflect on this research. For this purpose, 14 structured guideline interviews were conducted as a method of qualitative social research. The guideline interviews are transliterated, which is the data base for the following analyses. Hence, they will be evaluated with a qualitative content analysis. The first results regarding the terminology, suggestions for structural changes, and ethical evaluation of the scientists will be presented at TRIC 2021.

**Funding:** This joint research project “ONKOTHER-H” is supported by the European Social Fund (ESF), reference: ESF/14-BM- A55-0005/18, and the Ministry of Education, Science and Culture of Mecklenburg-Vorpommern, Germany.

### 2.18. On the Use of Plasma Activated Liquids for the Treatment of Cancer Cells

LauritaRomolo^1^,^2^,^7^BisagAlina^1^,^2^BucciCristiana^1^,^2^,^3^,^4^ColuccelliSara^1^,^2^,^3^,^5^,^6^GirolimettiGiulia^2^,^3^,^6^IacoPierandrea De^2^,^3^,^5^PerroneAnna Myriam^2^,^5^GherardiMatteo^1^,^2^,^7^MarchioLorena^2^,^3^,^6^PorcelliAnna Maria^2^,^4^,^8^TurriniEleonora^9^FimognariCarmela^9^ColomboVittorio^1^,^2^,^7^GasparreGiuseppe^2^,^3^,^6^1Department of Industrial Engineering2Centro di Studio e Ricerca sulle Neoplasie Ginecologiche3Department of Medical and Surgical Sciences4Department of Pharmacy and Biotechnology5Unit of Gynecologic Oncology, S. Orsola-Malpighi Hospital6Centre for Applied Biomedical Research7Interdepartmental Center for Industrial Research Advanced Mechanical Engineering Applications and Materials Technology8Interdepartmental Center for Industrial Research Life Sciences and Technologies for Health9Department for Life Quality Studies

In this work, a microsecond pulsed dielectric-barrier-discharge jet was used to produce PAL for the treatment of T-leukemia cells, spleen lymphoblast cell line, and normal lymphocytes, while a multiwire plasma source was used for the production of PAL for the treatment of Epithelial Ovarian Cancer (EOC) and non-cancer epithelial cell lines of ovarian origin (HOSE). On T-lymphoblastic cell line, PAL induced apoptosis through the activation of the intrinsic pathway and inhibited cell-cycle progression. The use of the scavengers NAC or O-phenantroline significantly decreased PAL pro-apoptotic activity. For the first time, results of PAL on leukemia cells cultivated in hypoxia, which plays a critical role in promoting chemoresistance, are presented. The PAL treatment showed a selective cytotoxic effect on EOC with respect to HOSE. Moreover, further investigation showed the ability of non-cancer cells to adapt to the oxidative burst, induced by PAL, by increasing antioxidant proteins (superoxide dismutase) levels. Taken together, our results provide a deeper understanding of the cellular and molecular impact of PAL on cancers cells, highlighting its partial selectivity towards malignant cells and its cytotoxic activity in the model of chemoresistance, such as cell cultured in hypoxia.

**Funding:** This work was supported by AlmaIDEA Senior Grant by Alma Mater Studiorum-Università di Bologna: “Chemo-physical and biological mechanisms behind the anticancer activity of plasma activated liquids for the treatment of peritoneal carcinosis from primitive epithelial ovarian/fallopian tube tumor” and by National SIR Grant (RBSI14DBMB) of the Italian Ministry of Education, Universities and Research, MIUR.

### 2.19. Plasma-Activated Medium-Mediated Endoplasmic Reticulum Stress Induced Selective Apoptosis of Mycobacterium Tuberculosis-Infected Macrophage ^§^

LeeChaebok^1^,^2^LeeKang In^1^,^2^KaushikNagendra^3^KimHwa-Jung^1^,^2^1Department of Microbiology, College of Medicine, Chungnam National University, Daejeon 35015, Korea2Biomedical Convergence Research Center, Chungnam National University, Daejeon 35015, Korea3Plasma Bioscience Research Center, Kwangwoon University, 447-1, Seoul 01897, Korea§This contribution was accepted but not part of the oral program.

Recently, plasma-activated medium (PAM) has been reported as a practical alternative to treating cancer lesions. It can also be used as an adjuvant therapy to remove cancel cells after surgery. Approximately 5% of pulmonary TB patients require surgery. If PAM selectively kills Mtb H37Rv (H37)-infected cells, it can be used as supportive treatment for tuberculosis lesions that require surgical treatment. However, to the best of our knowledge, there has not been any research into this yet. In this work, PAM was diluted in a cell culture medium with or without pyruvate (PYR) into concentrations that did not affect H37-uninfected macrophages. Then, we tested if this PAM can result in apoptosis of H37-infected cells through in vitro and in vivo mouse models. The result indeed shows apoptosis of H37-infected cells and a decrease in cell growth of intracellular bacteria. The apoptotic macrophages by PAM treatment downregulate caspase 3 and upregulate CHOP-related ER stress in H37-infected cells at the transcriptional level and Western blot. Moreover, the release of AIF from mitochondria was confirmed using fluorescence analysis. Our results show that the effect of OH radical and OH radical converted hydrogen peroxide generation in plasma plays an important role in selective apoptosis of H37-infected cells.

**Funding:** This study was supported by the National Research Foundation of Korea (NRF) funded by the Korean Government (MSIP) (No.2017R1A5A2015385), and partially by Brain Korea 21 PLUS Project for Medical Science, Chungnam National University School of Medicine.

### 2.20. Plasma Activated Water and Oncology Drugs: A Possible Combinatory Effect?

LopesBeatriz Pinheiro^1^O’NeillLiam^2^BourkePaula^1^,^3^,^4^BoehmDaniela^1^1Environmental Sustainability and Health Institute and School of Food Science and Environmental Health, Technological University Dublin, Dublin, Ireland2TheraDep Ltd., Clonmel, Ireland3Plasma Research Group, School of Biosystems and Food Engineering, University College Dublin, Dublin, Ireland4School of Biological Sciences, Queens University Belfast, Belfast, Northern Ireland, United Kingdom

Cancer is one of the major causes of death worldwide, with a need for alternative therapies that are effective against cancers resistant to conventional therapies and with lower side effects. Both cold atmospheric plasma and plasma-activated liquids, such as water (PAW), have been studied as a possible approach against cancer. However, the anti-cancer activity of PAW is dependent on the chemical compounds present in solution. For a safe clinical application, the active ingredients of PAW should be identified, their toxicity should be minimal for other cells and it is necessary to understand the possible combinatory effects with conventional therapies. The research aim is to investigate the therapeutic properties of a combination of different types of PAW and conventional oncology drugs. For combinatorial experiments with anti-cancer agents (such as topotecan), two types of plasma discharges are used to produce PAW with different chemical compositions. First, the IC50 values are determined for PAW and native drugs alone. Then, the anti-cancer effect of different combinations between PAW and oncology drugs are analyzed to establish possible additive, synergistic, or antagonistic effects. Finally, the effect obtained is characterized, trying to maximize cytotoxic activity on cancer cells, while minimizing the side effects obtained in normal cells.

**Funding:** This research is supported by funding from the Irish Research Council under the Enterprise Partnership Scheme and is performed in partnership with TheraDep Ltd.

### 2.21. Non-Thermal Plasma and Laser Irradiation as Cues That Bias Redox Balance in Cells

SmolkováBarbora^1^LunovaMariia^1^,^2^UzhytchakMariia^1^,^2^FrtúsAdam^1^KubinováŠárka^1^,^3^DejnekaAlexandr^1^LunovOleg^1^1Institute of Physics of the Czech Academy of Sciences, Prague, 18221, Czech Republic2Institute for Clinical & Experimental Medicine (IKEM), Prague, 14021, Czech Republic3Institute of Experimental Medicine of the Czech Academy of Sciences, Prague, 14220, Czech Republic

The emerging fields of non-thermal plasma (NTP) and low power lasers have shown considerable promise in various biomedical applications, including cancer therapy. However, understanding the molecular mechanisms procuring cellular responses remains incomplete. Indeed, the key to successful clinical transfer of any technology is a deep understanding of molecular mechanisms at the cell level.

Here, we would like to focus on potential mechanisms of action of NTPs and lasers, and challenges related to their identification. Firstly, we deciphered the molecular mechanisms of NTP-triggered cancer cell death. High levels of Bcl-2 protein expression resulted in cancer cell resistance to oxidative stress mediated by plasma. We disclosed detailed mechanisms of NTP-mediated alteration of redox signaling in liver cancer cells.

Secondly, we showed that mitochondria serve as a sub-cellular “sensor” and “effector” of laser light non-specific interactions with cells. Our findings reveal the mechanism of how laser irradiation interferes with cell homeostasis and underscore that such laser irradiation permits remote control of mitochondrial function in the absence of chemical or biological agents.

**Funding:** This research received no external funding.

### 2.22. The Amino Acid Metabolism Is Essential for Evading Physical Plasma-Induced Tumor Cell Death ^§^

MeyerDorothee^1^SagwalSanjeev Kumar^1^GandhirajanRajesh^1^,^2^BekeschusSander^1^1ZIK *plasmatis*, Leibniz Institute for Plasma Science and Technology (INP), Greifswald, Germany2Institute for Hygiene and Environmental Medicine, Greifswald University Medicine, Sauerbruchstr., Greifswald, Germany§This contribution was accepted but not part of the oral program.

Metabolic alterations foster adaptation to endogenous and exogenous cellular challenges and are regarded as a hallmark of cancer cells. However, the effect of metabolic reprogramming on cancer cells under gas plasma treatment remains to be clarified. Studying plasma resistant and sensitive cells under gas plasma treatment, we observed an increased utilization of metabolic substrates in resistant (HeLa, Panc-1) but not in sensitive cells (MeWo, OVCAR 3). Moreover, increased biosynthesis of amino acid transporter ASCT2 and chaperone CD98hc were detected in resistant cell lines. Cell viability under gas plasma treatment decreased following glutamine depletion, siRNA-mediated knockdown, or small molecule mediated inhibition of ASCT2 and increased following supplementation of glutamine, valine, or tyrosine in HeLa. In conclusion, amino acids involved in metabolic reprogramming impact cell death under gas plasma.

**Funding:** Funding was received by the German Federal Ministry of Education and Research (BMBF, grant numbers 03Z22DN11 and 03Z22Di1), the German Research Foundation (DFG, grant number AOBJ 669606), as well as the European Social Fund and the Ministry of Education, Science and Culture of Mecklenburg-Vorpommern, Germany (grant number ESF/14-BM-A55-0006-18). D.M. was supported by the Gerhard-Domagk-Foundation in Greifswald, Germany.

### 2.23. Cold Plasma-Treated Ringer’s Saline for the Treatment of Osteosarcoma

Mateu-SanzMiguel^1^,^2^,^3^TornínJuan^1^,^2^,^3^BrulinBénédicte^4^KhlyustovaAnna^1^,^2^,^3^GinebraMaria-Pau^1^,^2^,^3^,^5^LayrollePierre^4^CanalCristina^1^,^2^,^3^1Biomaterials, Biomechanics and Tissue Engineering Group, Dpt. Materials Science and Engineering, Escola d’Enginyeria Barcelona Est (EEBE), Technical University of Catalonia (UPC), Barcelona, Spain2Barcelona Research Center in Multiscale Science and Engineering, EEBE, UPC, Barcelona, Spain3Research Centre for Biomedical Engineering (CREB), UPC, Barcelona, Spain4Inserm, UMR 1238, PHY-OS, Laboratory of Bone Sarcomas and Remodeling of Calcified Tissues, Faculty of Medicine, University of Nantes, Nantes, France5Institute for Bioengineering of Catalonia (IBEC), Barcelona Institute of Science and Technology (BIST), c/Baldiri i Reixach 10-12, 08028 Barcelona, Spain

Osteosarcoma (OS) is the most common bone cancer and presents difficult treatment. The anti-cancer effects of cold atmospheric plasmas (CAP) have been proved in 2D OS cell cultures, related with the generation of RONS in culture media, but this model of study has poor clinical relevance. The aim of this work is to evaluate the effects of plasma-treated Ringer’s saline (PAR) in different OS models. For that, we used different OS cell lines (SaOS-2, MG-63 and U2-OS) and hBM-MSC in adherent culture and we also employed 3D cultures (consisting in a biomimetic material) and mouse tumor tissues. First, PAR reduced cell viability up to 20% and increased intracellular RONS (8-fold), DNA damage and apoptosis in OS cells, while sustained cell viability and fewer effects were determined in hBM-MSCs. Second, PAR decreased cell viability and expression of osteogenic markers in 3D cultures and Ki-67 expression in tumor tissues, but presenting increased resistance to the RONS generated in PAR than adherent cultures. To conclude, cytotoxicity of PAR is highly dependent on the concentration of RONS and the complexity of the in vitro model employed, being the intracellular RONS increase and consequent DNA damage key mediators of apoptosis. This study provides new insights to develop effective therapies based in CAP for OS.

**Funding:** This project has received funding from the European Research Council (ERC) under the European Union’s Horizon 2020 research and innovation programme (grant agreement No. 714793). Authors acknowledge the financial support of MINECO for PID2019-103892RB-I00/AEI/10.13039/501100011033, and of Generalitat de Catalunya for the 2017 SGR 1165, for the Scholarship of MMS 2019 FI-B00479 and for the support for the research of MPG and CC through the ICREA Academia Award for excellence in research.

### 2.24. Efficacy and Immunogenicity of Plasma-Oxidized Saline against Peritoneal Carcinomatosis In Vitro and In Vivo

MiebachLea^1^,^2^FreundEric^1^,^2^ClemenRamona^1^BekeschusSander^1^1ZIK *plasmatis*, Leibniz Institute for Plasma Science and Technology (INP), Greifswald, Germany2Department of General, Visceral, Thoracic, and Vascular Surgery, Greifswald University Medical Center, Greifswald, Germany

Gas plasma-oxidized liquids have received much attention lately due to their anticancer activities. Especially peritoneal carcinomatosis is a disease that might be targeted well using those liquids. One barrier of most studies is the utilization of liquids that are not clinically accepted. Here, 0.9% sodium chloride (NaCl), an accredited medical product was plasma-treated, and investigated for its anticancer effects. To estimate its stability, major long-lived species were quantified across several weeks of storage at −20 °C. Subsequently, its toxic effects against of human malignant cell lines of the peritoneal cavity was investigated in both 2D and 3D models, including the HET-CAM chicken embryo vascularized tumor model. In addition, co-cultures with human dendritic cells were performed to investigate the extent of immunogenic cell death (ICD) in tumor cells in response to these liquids. Finally, the efficacy of plasma-treated NaCl was tested in vivo. This study will complement existing work on plasma-oxidized liquids and suggests oxidized liquid to be effective adjuvant treatment options to target peritoneal carcinomatosis.

**Funding:** This research was funded by the Federal German Ministry of Education and Research (BMBF), grant numbers 03Z22DN11 and 03Z22Di1. Lea Miebach received support from the Gerhard-Domagk-Foundation.

### 2.25. Using Non-Thermal Plasma to Modulate Immunogenicity-Associated Markers in Leukemic T Lymphocytes

MohamedHager^1^ClemenRamona^2^FreundEric^2^LackmannJan-Wilm^2^,^3^WendeKristian^2^ConnorsJennifer^1^HaddadElias K.^4^DampierWill^1^GebskiEric^5^ReyesRufranshell^5^BeaneSamuel^5^StapelmannKatharina^6^WigdahlBrian^1^BekeschusSander^2^MillerVandana^1^1Institute for Molecular Medicine & Infectious Disease, and Department of Microbiology & Immunology, Drexel University College of Medicine, Philadelphia, Pennsylvania, United States of America2ZIK *plasmatis*, Leibniz Institute for Plasma Science and Technology (INP), Greifswald, Germany3CECAD proteomics facility, University of Cologne, Cologne, Germany4Division of Infectious Diseases and HIV Medicine, Drexel University College of Medicine, Philadelphia, Pennsylvania, United States of America5College of Arts and Sciences, Drexel University, Philadelphia, Pennsylvania, United States of America6Department of Nuclear Engineering, North Carolina State University, Raleigh, North Carolina, United States of America

T-cell acute lymphoblastic leukemia (T-ALL) is an aggressive cancer characterized by the accumulation of abnormal T-cells in the blood. Treatment involves chemotherapy with the risk of relapse partly due to drug resistance and low immunogenicity of leukemic cells that facilitates escape from an immune response. We investigated a novel approach for enhancing the immunogenicity of leukemic cells involving non-thermal plasma (NTP). NTP is an ionized gas that has documented immunomodulatory effects in vivo that were correlated with immune control of various solid tumor cancers. However, its therapeutic potential against hematologic diseases has not yet been investigated. We demonstrated that application of NTP to the Jurkat cell line model for leukemia induces cytotoxicity as well as emission of damage associated molecular patterns (DAMPs) that promote innate immune cell function. These include the release of IL-1 beta, the display of pro-phagocytic calreticulin (CRT), and display of heat shock proteins (HSP) 70 and 90. Additionally, NTP modulated molecules involved in antigen presentation and T-cell activation, and caused an altered array of peptides to be displayed on Jurkat cell surface MHC I. Together, these suggest the feasibility of using the immunomodulatory potential of NTP in an ex vivo strategy for stimulating immunity against T-ALL.

**Funding:** This research was supported by funds of the German Federal Ministry of Education and Research (BMBF, grant numbers 03Z22DN11, 03Z22DN12, and 03Z22Di1). This research was also facilitated by a travel grant provided by the Global Engagement Funding program of the Drexel University Office of Global Engagement and Education Abroad, as well as funding from the Department of Microbiology and Immunology and the Institute for Molecular Medicine and Infectious Disease at the Drexel University College of Medicine.

### 2.26. Cellular Effect of Pulsed High-Power Microwaves on Immunogenicity of Lungs Cancer Cells ^§^

RanaJuie^1^MumtazSohail^1^BhartiyaPradeep^1^KaushikNeha^2^NguyenLinh Nhat^1^KaushikNagendra Kumar^1^ChoiEun Ha^1^1Plasma Bioscience Research Center, Applied Plasma Medicine Center, Department of Plasma Bio Display, Department of Electrical and Biological Physics, Kwangwoon University, Seoul, South Korea2Department of Biotechnology, College of Engineering, University of Suwon, South Korea§This contribution was accepted but not part of the oral program.

This work aimed to investigate the effects of 3.5 GHz pulsed HPM radiation on lung cancer (A549 and H460) and normal (MRC5) cells. For exposure, 15 and 50 pulses are given to cells. Cell viability, metabolic activity, apoptosis, cell death, and reactive species were evaluated after the HPM exposure at low and high doses. It is observed that HPM exposure decreased the viabilities, metabolic activity in lung cancer A549 and H460 cells in a dose-dependent manner, while no significant effects on the lung fibroblast MRC5 cells. We have also investigated the effect of HPM exposure on immunogenic cell death phenomenon-related gene expression using lung cancer cells. Further studies will be focused on in depth underlying mechanisms induced by HPM exposure responsible for immunogenic cell death in cancer cells at indicated doses. Our findings show that the HPM could be a potential candidate for cancer cells responses compared to the lung fibroblast cells.

**Funding:** This research was funded by the National Research Foundation under the Ministry of Science and ICT (NRF-2016K1A4A3914113), (NRF-2021R1C1C1013875) of the Korean Government.

### 2.27. Anticancerous Plasma-Electro-Chemotherapy: First In-Vivo Studies

ChungThai-Hoa^1^SkliasKyriakos^2^StancampianoAugusto^3^GazeliKristaq^2^DarnyThibault^2^AndréFranck M.^1^DoziasSébastien^3^DouatClaire^3^PouvesleJean-Michel^3^SousaJoão Santos^2^RobertEric^3^MirLluis M.^1^1Université Paris-Saclay, CNRS, Institut Gustave Roussy, Metabolic and Systemic Aspects of Oncogenesis (METSY), Villejuif, France2Université Paris-Saclay, CNRS, Laboratoire de Physique des Gaz et des Plasmas, Orsay, France3GREMI, UMR 7344 CNRS/Université d’Orléans, Orléans, France

Following in vitro assessment of cancer cells and the benefit of a combined treatment based on incubation with plasma treated solution and application of Pulsed Electric Field in the context of reversible cell membrane permeabilization, this work reports on the in vivo evaluation of such combined innovative antitumor approach. Three independent studies, dealing with immunocompetent mice bearing fibrosarcoma tumors and involving control groups, PEF treated group (Bleomycine Electro Chemo Therapy (ECT) and combined plasma treated PBS (p-PBS) and PEF treatments, were performed. A single-shot treatment was applied 12 days after tumor cell injection, and follow up of tumor volume was performed for more than two months. The main conclusions of these studies indicate that (i) p-PBS production and storage was highly reproducible and allow for in vivo studies requiring large volume of such solutions; (ii) Bleomycin, p-PBS, and their combined injection has no antitumor action; (iii) ECT as a reference therapy is efficient, especially for fast growing tumors; (iv) combined p-PBS and ECT treatment allow for a slower tumor growth rate, for fast growing tumors, and lead to a significant increase of the number of tumor regressions; and (v) composition of p-PBS solution for different plasma exposures might be a unique parameter for a fine tuning of the efficiency of the combined antitumor treatment.

**Funding:** This research was funded by PLASCANCER project (INCa-PlanCancer n°17CP087-00) and with the support from GDR 2025 HAPPYBIO.

### 2.28. Combination Therapy with Cold Physical Plasma and Novel Molecules Using the Cutaneous and Squamous Melanoma In Vitro

SagwalSanjeev KumarBekeschusSanderZIK *plasmatis*, Leibniz Institute for Plasma Science and Technology (INP), Greifswald, Germany

Despite advances in chemotherapy and immunotherapy, the success in drug treatment in multiple tumors remains limited due to side effects and resistance to treatment due to immunoediting or metabolic adaptation of the tumor cells. Thus, it is necessary to search for new strategies that can prevent cancer relapse and increase the patient’s survival and quality of life. In the past few years, there has been a revolution in the use of small inhibitory molecules to treat cutaneous melanoma, which shows a significant increase in patient overall survival. These novel molecules have shown various biological and cytotoxic effects based on the inhibition of protein kinases which are essential for controlling the cell cycle. Cold plasma is a partially ionized gas that proved its effectiveness for different health care and medical applications. The partially ionized gas generates reactive oxygen and nitrogen species deposited in cell culture media to kill cancer cells by various mechanisms, such as increased the expression of specific transporters, which promotes the influx of the drug inside the cancer cells without affecting the efflux channels. These reactive species target the mitochondrial network. A 3D tumor spheroid screening was performed and is presented that highlights several new molecules acting in concert with medical gas plasma technology to promote skin cancer cell death.

**Funding:** This research was funded by the European Social Fund (ESF) and the Ministry of Education, Science, and Culture of Mecklenburg-West Pomerania, Germany, grant number ESF/14-BM-A55-0006/18, as well as the German Federal Ministry of Education and Research (BMBF), grant numbers 03Z22DN11 and 03Z22Di1.

### 2.29. DNA Toxicity and Mutagenicity of Cold Atmospheric Pressure Plasma and Small Molecules in Skin Cancer Treatment

SchäferMirijam^1^SemmlerMarie Luise^1^BernhardtThoralf^1^GlatzelAnnika^1^FreyAnna^2^SagwalSanjeev^3^HeinMartin^2^LangerPeter^2^KaufmannDieter^4^von WoedtkeThomas^3^BekeschusSander^3^FischerTobias^1^EmmertSteffen^1^BoeckmannLars^1^1Clinic and Polyclinic for Dermatology and Venereology, University Medical Center Rostock, Rostock, Germany2Institute of Chemistry, University Rostock, Rostock, Germany3ZIK *plasmatis*, Leibniz Institute for Plasma Science and Technology (INP), Greifswald, Germany4Institute of Organic Chemistry, Clausthal University of Technology, Clausthal, Germany

The potential use of cold atmospheric pressure plasma (CAP) for cancer treatment alone or in combinational therapies has gained increasing interest. While several studies have reported DNA double strand breaks as a consequence of CAP treatment, little is known regarding other potentially CAP-induced DNA lesions. In this project we investigate the toxicity of CAP and novel small molecules as well as the combination of both with focus on DNA damage. The inhibitory doses at which 50 percent of cells die have been determined using a cutaneous squamous cell carcinoma cell line and non-malignant keratinocytes. Treatment with small molecules alone had no effect on cell cycle distribution or induction of apoptosis or necrosis. Scratch assays, however, revealed an inhibitory effect of these substances on cell migration. Immunofluorescence assays are used to quantify DNA-crosslinks, pyrimidine dimers, oxidative lesions, and DNA double strand brakes. Furthermore, the DNA repair capacity of the cells is assessed using a host cell reactivation assay and compared to cellular toxicity of the respective treatment. Results from this project as part of a larger consortium will help to further untangle the molecular mechanisms for the efficacy of these innovative anti-cancer therapies.

**Funding:** This joint research project “ONKOTHER-H” is supported by the European Social Fund (ESF), reference: ESF/14-BM-A55-0001/18, 04/18 & 06/18, and the Ministry of Education, Science and Culture of Mecklenburg-Vorpommern, Germany. S.E. is supported by the Damp Stiftung. S.B. is supported by the German Federal Ministry of Education and Research (BMBF), grant number 03Z22DN11.

### 2.30. Plasma Treatment of Intraoral Mucosal Disorders: Evaluation of Safety

SeebauerChristian^1^BekeschusSander^2^von WoedtkeThomas^2^WeltmannKlaus-Dieter^2^MetelmannHans-Robert^1^1Department of Oral and Maxillofacial Surgery/Plastic Surgery, University Medicine Greifswald, Greifswald, Germany2ZIK *plasmatis*, Leibniz Institute for Plasma Science and Technology (INP), Greifswald, Germany

The kINPen MED has been approved as a medical device since 2013 and has been used since 2016 to treat various intraoral mucosal diseases in the Department of Oral and Maxillofacial Surgery at the University Medicine Greifswald. This is an assessment of safety and efficacy. In the period from 2016 until now, patients suffering from inflammatory mucosal diseases (*n* = 14, age from 23 to 86 years), intraoral wound healing disorders (*n* = 7, age from 43 to 75 years), as well as leukoplakia (*n* = 6, age from 36 to 68 years), and oral lichen planus (*n* = 16, age from 54 to 80 years), known to be premalignant lesions, have been treated with cold plasma (kINPen med, neoplasm tools, Greifswald, Germany) and assessed for efficacy, side effects, and potential carcinogenesis. Of these 43 patients, one patient was treated 25 times (12 months), one patient 34 times (14 months), one patient 38 times (6 months), and one patient even 70 times in 36 months. Each patient was periodically reevaluated to assess possible changes in the mucosa. Wound healing was assisted and accelerated in all patients with intraoral wound healing disorders. In the treatment of leukoplakia, in some cases, a reduction was achieved. In the treatment of oral lichen planus and inflammatory mucosal diseases, in 70% of cases a relief and a reduction of the inflammation could be achieved. Except for pain in the area of exposed tooth necks, no side effects occurred. No patient developed suspicious mucosal changes or cancer, either during plasma treatment or during the follow-up period. In conclusion, cold plasma is particularly effective in inflammatory mucosal disorders and can be considered safe for treating mucosal disorders.

**Funding:** This work is mainly funded by the Greifswald University Medical Center (Greifswald, Germany) and co-sponsored by basic funds of the Leibniz Institute for Plasma Science and Technology (INP; Greifswald, Germany).

### 2.31. Non-Thermal Plasma-Guided Modulation of Immune Cells. Challenges and Perspectives

SmolkováBarbora^1^FrtúsAdam^1^UzhytchakMariia^1^LunovaMariia^1^,^2^KubinováŠárka^1^DejnekaAlexandr^1^LunovOleg^1^1Department of Optical and Biophysical Systems, Institute of Physics of the Czech Academy of Sciences, Prague, Czech Republic2Institute for Clinical & Experimental Medicine (IKEM), Prague, Czech Republic

In recent years, a large number of studies of non-thermal plasma (NTP) have progressed to a level that makes it feasible to specifically design, manufacture, and characterize NTP suitable for clinical applications. Such a burst of research activities on NTP demonstrated a fast evolution and a great potential of this new interdisciplinary field. It is worth noting here, that despite years of extensive research, the mechanisms of non-thermal plasma-induced biological effects are still not fully understood. Indeed, NTP cellular targets as well as the molecular or biophysical fundamentals of its alleged biological effects remain generally unknown. It is widely accepted that plasma-generated reactive oxygen and nitrogen species (RONS) can indirectly modulate the cells. Depending on the concentration, RONS act as activators or suppressors of immunity, inflammation, or carcinogenesis. In general, the manipulation of RONS balance can be a potential therapeutic approach not only in cancer. Recently, a growing number of studies are showing the potential immunomodulatory effects of NTP. However, the application of NTP is relatively new approach in biomedicine and needs to prove if it can meet the high expectations in evidence-based medicine-controlled health care systems.

**Funding:** This research was funded by Operational Programme Research, Development and Education financed by the European Structural and Investment Funds and the Czech Ministry of Education, Youth and Sports (Project No. SOLID21-CZ.02.1.01/0.0/0.0/16_019/0000760), and by the Ministry of Industry and Trade of the Czech Republic: FV10081.

### 2.32. DNA Damage Induced by Plasma: Opportunities in Cancer Therapy and Safety Implications

SziliEndre^1^GaurNishtha^2^KuritaHirofumi^3^OhJun-Seok^4^HongSung-Ha^5^FukuharaHideo^6^ShortRob^2^1University of South Australia, Adelaide, Australia2Lancaster University, Lancaster, United Kingdom3Toyohashi University of Technology, Toyohashi, Japan4Osaka City University, Osaka, Japan5Australian National University, Canberra, Australia6Kochi Medical School, Kochi, Japan

Plasma is an exciting a new technology being intensively researched across many areas in biology and medicine. Depending on the area of application and the method of plasma treatment employed, plasma can induce destructive effects (e.g., cell death) or stimulatory effects (e.g., cell growth). The contrasting effects of plasma can be beneficial for applications involving the eradication of cancer cells or pathogenic microorganisms for stimulation of tissue healing. Although the broad spectrum of plasma effects provides significant opportunities in healthcare, it also poses a potential significant risk through unwanted damage to healthy cells and tissue. This risk is significant because plasma readily produces highly reactive molecules, and in particular the hydroxyl radical, which are known to readily cause DNA damage in cells. Therefore, it is important to continually evaluate the potential safety risks of plasma and to design strategies to mitigate these risks. In this presentation I will cover experiments and assays we have employed to assess DNA damage caused by plasma treatments and a method that can be used to prevent DNA damage in cells for applications where DNA damage is not desired.

**Funding:** ES acknowledges support from the Australian Research Council through the Future Fellowship FT190100263 and National Health Medical Research Council Ideas Grant 2002510. JSO acknowledges support from Osaka City University (OCU) Strategic Research Grant 2019 for Top Priority Researches and BioMedical Engineering Center (BMEC). HF acknowledges JSPS/MEXT KAKENHI number JP19K18564.

### 2.33. Application of Plasma Activated Medium in Dendritic Cell-Based Cancer Vaccines

TomićSergej^1^PetrovićAnđelija^2^PuačNevena^2^ŠkoroNikola^2^BekićMarina^1^PetrovićZoran^2^VučevićDragana^1^ČolićMiodrag^1^,^3^,^4^1Department for Immunology and Immunoparasitology, Institute for the Application of Nuclear Energy, University in Belgrade, Belgrade, Serbia2Institute for Physics, University in Belgrade, Belgrade Serbia3Serbian Academy for Sciences and Arts, Belgrade, Serbia4Medical Faculty Foča, University of East Sarajevo, Foča, Bosnia and Hercegovina

Dendritic cells (DCs)-based vaccines emerged as quite promising for active immunotherapy of cancer, particularly in cancers that display poor immunogenicity. However, clinical efficacy of DC vaccines in some cancers is quite low, mostly due to adverse suppressive properties of tumor lysates used in autologous DC vaccines as a source of tumor antigens. We found that plasma activated medium (PAM) eliminates adverse suppressive effects of tumor lysates in DC vaccines and improves the anti-tumor functions of DCs in vitro. Namely, tumor cells treated with PAM displayed accumulation of reactive oxygen species (ROS), increased membrane expression of heath-shock proteins, release of IL-1b, and cell death by apoptosis and necrosis. DCs loaded with PAM-treated tumor lysates displayed an increased maturation potential, as judged by their higher expression of CD83, CD40, IL-12/IL-10 ratio production, and lower expression of IL-10, PDL1, and ILT-4. Moreover, in co-culture with autologous and allogeneic T cells, DC-loaded with PAM-treated lysates stimulated T cell proliferation and increased the proportion of cytotoxic CD8+ T cells and IL-17A-producing T cells, respectively. Additionally, the frequency of Th2 cells and regulatory CD4 and CD8 T cell subtypes was significantly lower than in co-cultures with control tumor lysates-loaded DCs. Cumulatively, these results suggest that the novel method for preparation of immunogenic tumor lysates with PAM could be suitable for improved DC-based immunotherapy of cancer patients.

**Funding:** This research was funded by the Ministry of Education, Science and Technological Development, grant no. 451-03-68/2020-14/200019, 451-03-68/2020-14/20024, Serbian Academy of Sciences and Arts grant No. F31.

### 2.34. Investigation of the Mechanistic and Immunogenic Response of Auranofin Combined with Cold Atmospheric Plasma in Glioblastoma

LoenhoutJinthe Van^1^BoullosaLaurie Freire^1^QuatannensDelphine^1^PeetersMarc^1^,^2^BogaertsAnnemie^3^SmitsEvelien^1^DebenChristophe^1^1Center for Oncological Research (CORE), Integrated Personalized & Precision Oncology Network (IPPON), University of Antwerp, Wilrijk, Belgium2Department of Oncology, Multidisciplinary Oncological Center Antwerp, Antwerp University Hospital, Edegem, Belgium3Plasma Lab for Applications in Sustainability and Medicine ANTwerp (PLASMANT), University of Antwerp, Wilrijk, Belgium

Oxidative stress has recently been highlighted as promising target for anticancer strategies. Targeting the redox balance of glioblastoma (GBM) cells by inducing high oxidative stress through delivery of exogenous reactive oxygen species (ROS) and inhibiting endogenous protective antioxidant system might be a beneficial and promising GBM treatment strategy. We investigated the mechanistic and immunogenic response after combination treatment with cold atmospheric plasma to induce exogenous ROS, and auranofin, a thioredoxin reductase inhibitor, in 2D and 3D GBM cell cultures. Sequential combination treatment of auranofin and cold atmospheric plasma resulted in a synergistic response in 2D and 3D GBM cell cultures. We confirmed a ROS-mediated response, which was able to induce distinct cell death mechanisms, specifically apoptosis and ferroptosis. Combination treatment-induced cell death resulted in an increased release of danger signals (ecto-calreticulin, ATP, and HMGB1) and DC maturation, indicating a potential increase in immunogenicity, though, the phagocytotic capacity of dendritic cells was inhibited by auranofin. In vivo optimization and validation of this combination strategy in GBM is currently ongoing. In conclusion, our study provides a novel therapeutic strategy for GBM to enhance the efficacy and immunogenicity of oxidative stress-inducing therapy through a combination of auranofin and cold atmospheric plasma.

**Funding:** This research was funded by the Olivia Hendrickx Research Fund (21OCL06) and the University of Antwerp (FFB160231).

### 2.35. Tailoring the Chemistry of Plasma Treated Water Solutions to Selectively Attack Cancer Cells

VeronicoValeria^1^GristinaRoberto^2^FaviaPietro^1^,^2^FracassiFrancesco^1^,^2^SardellaEloisa^2^1Dipartimento di Chimica, Università degli Studi di Bari Aldo Moro, via Orabona, 4, 70126 Bari, Italy2CNR- Istituto di Nanotecnologia (CNR-NANOTEC) UoS Bari, c/o Dipartimento di Chimica, Università degli Studi di Bari Aldo Moro, via Orabona, 4, 70126 Bari, Italy

Plasma Treated Water Solutions (PTWS) recently proved to be efficacious system for the delivery of Reactive Oxygen and Nitrogen Species (RONS) to biological targets with promising results for cancer treatment. In most of in vitro experiments PTWS are produced by plasma treatment of biocompatible liquids like cell culture media. In spite of quite clear evidence of anti-cancer effects, however, the chemistry generated in PT-cell culture media is rarely investigated due to the presence of more than 45 components among amino acids, vitamins, carbohydrates, and inorganic salts severely complicating the study of such systems. Recent studies, though, strongly attest for the need to precisely tailor the chemistry of PT-cell culture media to selectively attack cancer cells. In this presentation, therefore, we discuss the most critical aspect in the modulation and analysis of RONS in PT-cell culture media to achieve the ideal chemical composition to selectively attack the type of cancer cells under investigation. In particular, we will show that the concentration of nitrite anions (NO_2_^−^) can be optimized in the liquids to selectively decrease the tolerance threshold for H_2_O_2_ in osteosarcoma cancer cells as well as on colorectal adenocarcinoma with respect to endothelial hybrid cells.

**Funding:** Regione Puglia: under grants “LIPP” (grant no. 51, within the Framework Programme Agreement APQ “Ricerca Scientifica”, II atto integrative—Reti di Laboratori Pubblici di Ricerca).

### 2.36. Selective Inhibition of Adhesion of Dermal Tumor Cells by Plasma-Activated Medium

WaldnerAnna-C.^1^BergemannClaudia^1^ReblHenrike^1^BoeckmannLars^2^EmmertSteffen^2^NebeJ. Barbara^1^1Dept. of Cell Biology, Rostock University Medical Center, Rostock, Germany2Clinic and Polyclinic for Dermatology and Venerology, Rostock University Medical Center, Rostock, Germany

Use of cold atmospheric pressure plasma (CAP) provides a novel strategy to treat skin cancers. Cutaneous squamous cell carcinoma has an increasing incidence worldwide. We determined whether plasma-activated medium (PAM) affects the adhesion capacity of dermal carcinoma cells. The squamous cell carcinoma cell line A431 and non-malignant keratinocytes (HaCaT) were cultured under the same conditions in Dulbecco’s Modified Eagle Medium (DMEM). PAM was generated from DMEM without serum using the kINPen 09 (Ar, 1.9 slm) [3]. The cell adhesion was recorded online via an impedance-based method (xCELLigence RTCA S16). In general, PAM has an inhibitory effect on cell adhesion. Cell adhesion was markedly reduced in A431 cells compared to HaCaT cells. The actin cytoskeleton is important for cell adhesion as well as shape, spreading and migration. PAM decreased the sub-membranous localization of the actin cytoskeleton in A431, which is characteristic for epithelial cells. In contrast, PAM did not change the organization of actin in HaCaT control cells. Quantification of actin cytoskeleton parameters, such as number and length of actin filaments, was conducted using FilaQuant software. We conclude that PAM has a selective inhibitory effect on both adhesion and actin cytoskeleton organization in dermal carcinoma cells compared with non-malignant keratinocytes.

**Funding:** This project “ONKOTHER-H” is supported by the European Social Fund (ESF/14-BM-A55-0002/18), and the Ministry of Education, Science and Culture of Mecklenburg-Vorpommern, Germany.

### 2.37. Comparison of 2D and 3D Human Glioblastoma Multiforme Cell Culture Models for Cold Atmospheric Plasma Induced Cytotoxicity

WanigasekaraJanith^1^,^2^,^3^CullenPatrick J.^1^,^5^,^6^TiwariBrijesh^2^CurtinJames F.^1^,^3^,^4^1BioPlasma Research Group, School of Food Science and Environmental Health, Technological University Dublin, Dublin, Ireland2Department of Food Biosciences, Teagasc Food Research Centre, Ashtown, Dublin, Ireland3Environmental Sustainability & Health Institute (ESHI), Technological University Dublin, Dublin, Ireland4FOCAS Research Institute, Technological University Dublin, Dublin, Ireland5University of Sydney, School of Chemical and Biomolecular Engineering, Sydney, Australia6Plasmaleap Technologies, Merewether Building, City Road, Sydney, Australia

Three-dimensional (3D) cells that maintain physiological cell–cell and cell–extracellular matrix interactions, more closely mimic the natural in vivo environment. This provides accurate representation of plasma induced toxicological resistance, cellular responses, and gene expression. This study compared novel pin-to-plate cold atmospheric plasma (CAP) device induced cytotoxicity of U-251MG human glioblastoma multiforme (GBM) in 3D and 2D cell cultures. U-251MG tumorsphere (low attachment plates—Nunclon™ Sphera™) and 2D cells were constructed, then dose–response curves were established by exposing cells to six different doses of CAP (20–320 s) at 240 V, 1000 Hz, and 73 µs (duty cycle). After 24 h incubation at 37 °C the cell viability was measured using Alamar Blue™ assay. An IC50 of 160.4 s (157.0 s ± 163.9 s) and 386.3s (375.9 s ± 397.1 s) were found for 2D cells and 3D cells, respectively. The viability of the U-251MG 3D cells was decreased, but they showed higher plasma induced cytotoxicity resistant compared to 2D-cultured cells. In conclusion, the pin-to-plate CAP device successfully induced GBM cell death in a dose dependent manner, and also 3D cell culture as better alternative to overcome the cons of in vivo animal testing and conventional 2D cell culture by providing a more accurate platform for CAP application in GBM therapy.

**Funding:** The project has been supported by Science Foundation Ireland (SFI) under Grant Number 17/CDA/4653.

### 2.38. Investigation of Small Molecules in Combination with Cold Atmospheric Plasma as Innovative Therapies for the Treatment of Melanoma and Squamous Cell Carcinoma

WendtFranziskaThammElisabethHamannBiancaRamerRobertHinzBurkhardInstitute of Pharmacology and Toxicology, Rostock University Medical Center, Rostock, Germany

This project focuses on the anticarcinogenic activity of small molecules alone and in combination with cold atmospheric pressure plasma (CAP) on melanoma (A375) and squamous cell carcinoma cells of the skin (A431). Thereby, 3-[3′-Oxo-benzo[b]thiophen-2′-(Z)-yliden]-1-(β-D-glucopyranosyl)-oxindol (KD87), a thio-analogous indirubin-N-glycoside, was found to induce proapoptotic and viability-reducing effects. The cytotoxic effect of KD87 was enhanced by concomitant treatment with CAP. Furthermore, the angiogenic capacities of human endothelial cells were inhibited when exposed to the conditioned medium of KD87-treated cancer cells, indicating an additional antiangiogenic effect of KD87. Further small molecules also shown to have viability-reducing effects on A375 and A431 cells were 3-[3′-Oxo-benzo[b]thiophen-2′-(Z)-yliden]-1-(β-D-mannopyranosyl)-oxindol (KD85), another indirubin-N-glycoside, and the phytocannabinoids Δ9-tetrahydrocannabinol (THC) and cannabidiol (CBD). The cytotoxic effects of KD87, THC, and CBD were accompanied by induction of the enzyme heme oxygenase-1 (HO-1), with a functional role of this induction currently being tested. In addition, extracellular and intracellular quantification of KD87 using LC-MS/MS will allow us to determine in which cellular compartments KD87 becomes incorporated in the course of its cytotoxic action. In summary, it is expected that the results from this approach, together with those from other subprojects of ONKOTHER-H, will provide a development platform for further innovative anti-cancer therapies.

**Funding:** The joint research project “ONKOTHER-H” is supported by the European Social Fund (ESF), reference: ESF/14-BM-A55-0002/18, and the Ministry of Education, Science and Culture of Mecklenburg-Vorpommern, Germany.

### 2.39. Physical Effect of CAP on Cancer Cells

YanDayunKeidarMichaelDepartment of Mechanical and Aerospace Engineering, George Washington University, Washington, DC, 20052, United States of America

Cold atmospheric plasma (CAP), an ionized gas, composed of complex multi chemical and physical factors, has shown promising application in cancer treatment. Over the past decade, significant progress has been made in this direction. Both chemical and physical factors in CAP have been demonstrated to have unique biological impacts on cancer cells. From a chemically based vision, the anti-cancer efficacy will be completely determined by the cellular sensitivity to chemical factors in CAP, particularly reactive species. However, such a nature just sets a limitation for the efficacy of CAP, because some cancer cells are resistant to reactive species particularly H_2_O_2_. In contrast, physical factors in CAP may be mainly electromagnetic effect, can also impact cancer cells under specific experimental conditions, which not only causes a strong killing effect on some reactive species-resistant cell lines but also provide non-invasive potential to use CAP in clinic application. Here, we will focus on two physical effects: direct killing by physically based strategy and sensitization of cancer cells to widely used drugs. We hope these discussions could inspire peers in terms of thinking new directions in CAP-based cancer therapy.

**Funding:** This research was funded by National Science Foundation grant 1747760.

